# Left atrial strain assessment using cardiac computed tomography in patients with hypertrophic cardiomyopathy

**DOI:** 10.1007/s11604-023-01401-6

**Published:** 2023-02-22

**Authors:** Takaaki Hosokawa, Hiroshi Kawakami, Yuki Tanabe, Naoki Fukuyama, Kazuki Yoshida, Kentaro Ohara, Takuya Kitamura, Naoto Kawaguchi, Tomoyuki Kido, Takayuki Nagai, Katsuji Inoue, Osamu Yamaguchi, Teruhito Kido

**Affiliations:** 1 Department of Radiology, Ehime University Graduate School of Medicine, Shitsukawa, Toon, Ehime 791-0295 Japan; 2 Department of Cardiology, Pulmonology, Hypertension and Nephrology, Ehime University Graduate School of Medicine, Shitsukawa, Toon, Ehime 791-0295 Japan

**Keywords:** Cardiac computed tomography, Left atrial strain, Hypertrophic cardiomyopathy

## Abstract

**Purpose:**

To evaluate left atrial (LA) function in patients with hypertrophic cardiomyopathy (HCM) by LA strain assessment using cardiac computed tomography (CT-derived LA strain).

**Materials and methods:**

This was a retrospective study of 34 patients with HCM and 31 non-HCM patients who underwent cardiac computed tomography (CT) using retrospective electrocardiogram-gated mode. CT images were reconstructed every 5% (0–95%) of the RR intervals. CT-derived LA strain (reservoir [LASr], conduit [LASc], and booster pump strain [LASp]) were semi-automatically analyzed using a dedicated workstation. We also measured the left atrial volume index (LAVI) and left ventricular longitudinal strain (LVLS) for the left atrial and ventricular functional parameters to assess the relationship with CT-derived LA strain.

**Results:**

CT-derived LA strain significantly correlated with LAVI: *r* = − 0.69, *p* < 0.001 for LASr; *r* = − 0.70, *p* < 0.001 for LASp; and *r* = − 0.35, *p* = 0.004 for LASc. CT-derived LA strain also significantly correlated with LVLS: *r* = − 0.62, *p* < 0.001 for LASr; *r* = − 0.67, *p* < 0.001 for LASc; and *r* = − 0.42, *p* = 0.013 for LASp. CT-derived LA strain in patients with HCM was significantly lower than that in non-HCM patients: LASr (20.8 ± 7.6 vs. 31.7 ± 6.1%, *p* < 0.001); LASc (7.9 ± 3.4 vs. 14.2 ± 5.3%, *p* < 0.001); and LASp (12.8 ± 5.7 vs. 17.6 ± 4.3%, *p* < 0.001). Additionally, CT-derived LA strain showed high reproducibility; inter-observer correlation coefficients were 0.94, 0.90, and 0.89 for LASr, LASc, and LASp, respectively.

**Conclusion:**

CT-derived LA strain is feasible for quantitative assessment of left atrial function in patients with HCM.

## Introduction

Hypertrophic cardiomyopathy (HCM), which is the most common hereditary non-ischemic cardiomyopathy, occurs in 1 in 200–500 people [1, 2]. Hypertrophy, fibrosis, and disarray of the left ventricular (LV) myocardium cause diastolic dysfunction in HCM [3]. LV diastolic dysfunction is related to elevated LV filling pressure, which causes left atrial (LA) dysfunction via elevated LA pressure and fibrosis of the LA wall. Impaired LA function is associated with adverse outcomes, such as cardiovascular mortality, congestive heart failure hospitalization, cerebrovascular disease, and atrial fibrillation (AF) in patients with HCM [4]. Although the life expectancy of patients with HCM is comparable to that of the healthy population with appropriate therapeutic intervention, these complications affect patient mortality and the quality of life [5]. Therefore, LA function evaluation is important for risk stratification in patients with HCM.

LA size (e.g., diameter and dimension) and volume are conventional quantitative parameters for LA function [4]. However, LA function consists of three components: reservoir function, conduit function, and booster pump function; these conventional parameters are insufficient for assessing complex LA function [6, 7]. Recently, LA strain using speckle tracking echocardiography (STE) has been used to evaluate LA function. Even in patients without LA enlargement, LA strain has already decreased, and is associated with various adverse outcomes [8, 9]. Thus, LA strain is a more sensitive marker compared to conventional parameters for assessing LA function. However, several challenges exist in echocardiographic LA strain assessment owing to deep attenuation, limited acoustic window, and examiners’ technique causing impaired image quality [10–12]. Cardiac magnetic resonance imaging (MRI) is another modality used for LA strain assessment [8]. Although it is the gold standard for cardiac functional evaluation, several contraindications exist, including claustrophobia and metallic devices. Cardiac computed tomography (CT) is a widely used modality for evaluating coronary artery disease. Cardiac CT has high spatial resolution, without any blind spot, and high objectivity, independent of the examiners’ technique. Additionally, it allows accurate LV and LA morphological and functional assessment [13, 14]. LA strain assessment using cardiac CT (CT-derived LA strain) has emerged as a novel quantitative parameter for LA function [11, 12, 15]. However, the feasibility of CT-derived LA strain has not been fully evaluated in patients with HCM. In this study, we aimed to assess the LA function using CT-derived LA strain in patients with HCM.

## Materials and methods

### Study population

This retrospective study was approved by the local institutional review board (Registration number: 2106011), which waived the need for informed consent.

**Fig. 1:**
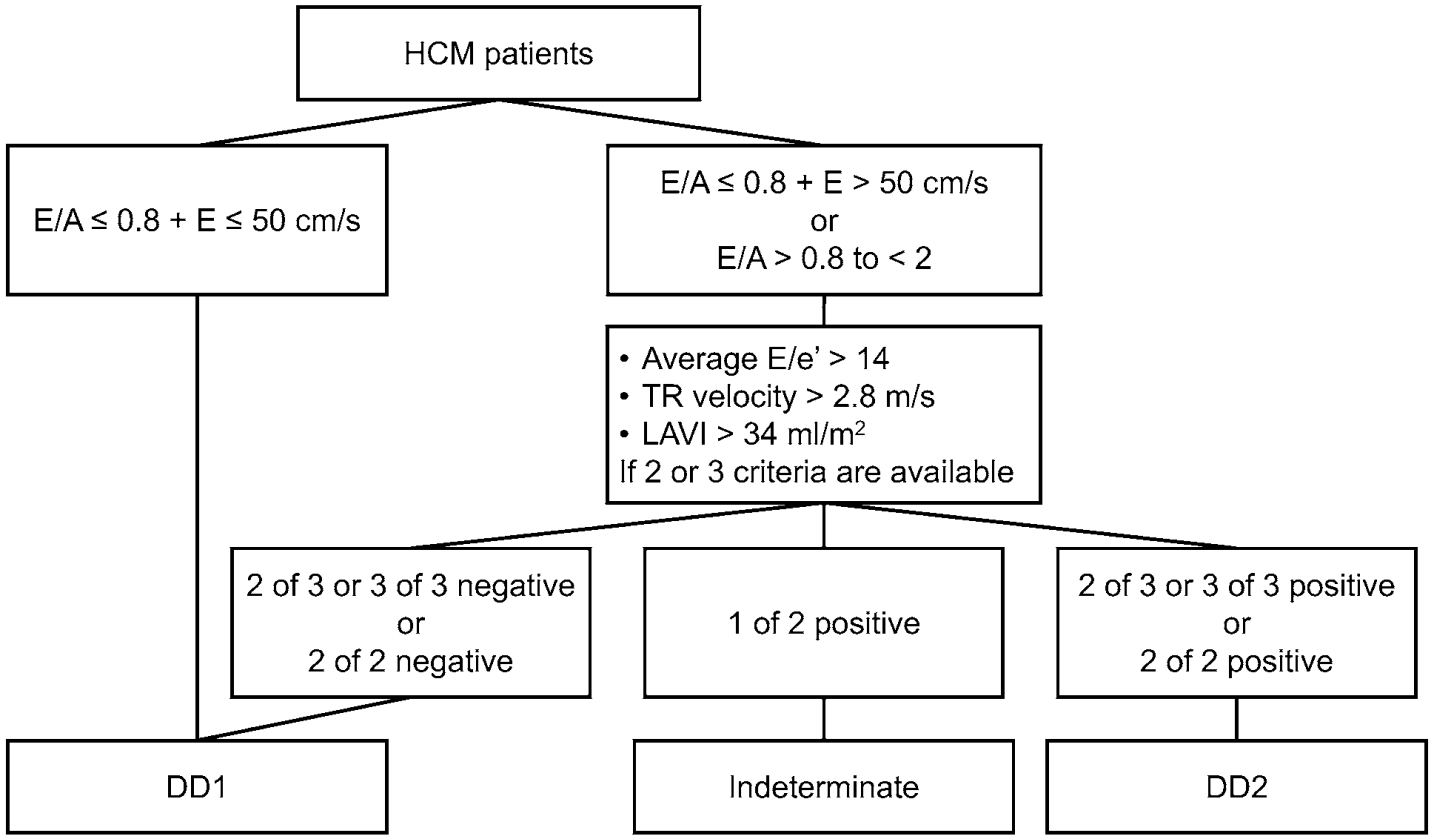
Algorithm for echocardiographic estimation of diastolic dysfunction grade in patients with HCM. *HCM* hypertrophic cardiomyopathy, *TR velocity* tricuspid valve regurgitant velocity, *LAVI* left atrial volume index, *DD1* Grade 1 diastolic dysfunction, *DD2* Grade 2 diastolic dysfunction

Using our radiology reporting database, we searched for patients with HCM who underwent cardiac CT using retrospective electrocardiogram (ECG)-gated mode without beta-blocker administration between March 2016 and June 2022. All the patients were diagnosed with HCM by cardiologists based on the JCS/JHFS guidelines [16]. The following patients were excluded: (1) patients who were not in sinus rhythm at the time of cardiac CT scanning, and (2) patients with a pacemaker (PM) or an implantable cardioverter-defibrillator (ICD). To compare the CT-derived LA strain between patients with HCM and non-HCM patients, patients without arrhythmia, cardiomyopathy, congenital heart disease, atherosclerotic cardiac disease, any history of cardiac surgery, or any medications that affect the cardiac function were selected as non-HCM patients based on electronic medical records, cardiac CT, and echocardiographic findings [17]. LV diastolic dysfunction grade was evaluated based on echocardiographic findings (Fig. 1) [18].

### Cardiac CT scan and post-processing

Cardiac CT was performed using a third-generation dual-source CT scanner (SOMATOM Force; Siemens Healthineers, Erlangen, Germany) and an automatic dual injector (Stellant DualFlow; Bayer Yakuhin, Osaka, Japan). All patients, except those with hypertrophic obstructive cardiomyopathy (HOCM), received 0.6 mg of nitroglycerin (Myocor; Astellas Pharma, Tokyo, Japan). To assess the scan timing and dilution rate of the contrast agent for cardiac CT, a 20% diluted contrast agent (Iopamidol 370 mg iodine/ml; Bayer Yakuhin, Osaka, Japan) was injected according to the modified timing bolus scan [19]. Cardiac CT was performed with contrast agent injection based on the results of the timing bolus scan. All the cardiac CT scans were performed using retrospective ECG-gated mode. The scan parameters were as follows: tube voltage, 90–120 kVp adjusted to body mass index; tube current, 40–478 mAs (reference mAs, 216 mAs); ECG dose modulation with full radiation exposure at 45% (heart rates [HR] ≥ 75 beats per minute [bpm]) or 75% (HR < 75 bpm) of the RR interval, with the low-mAs acquisition (25% of full dose) for the other cardiac phases; rotation time, 250 ms; pitch factor, 0.15; slice collimation, 2 × 192 × 0.6 mm. Cardiac CT data were reconstructed in 5% increments of the RR interval throughout the cardiac cycle (0–95%, 20 phases), with a slice thickness and increment of 0.75 mm and 0.4 mm, respectively. Medium soft convulsion kernel (Siemens Bv40) and model-based iterative reconstruction (ADMIRE, Siemens Healthineers, Erlangen, Germany) at a strength level of two were used [17].

### LA and LV function assessment by CT imaging

#### LA strain analysis

**Fig. 2:**
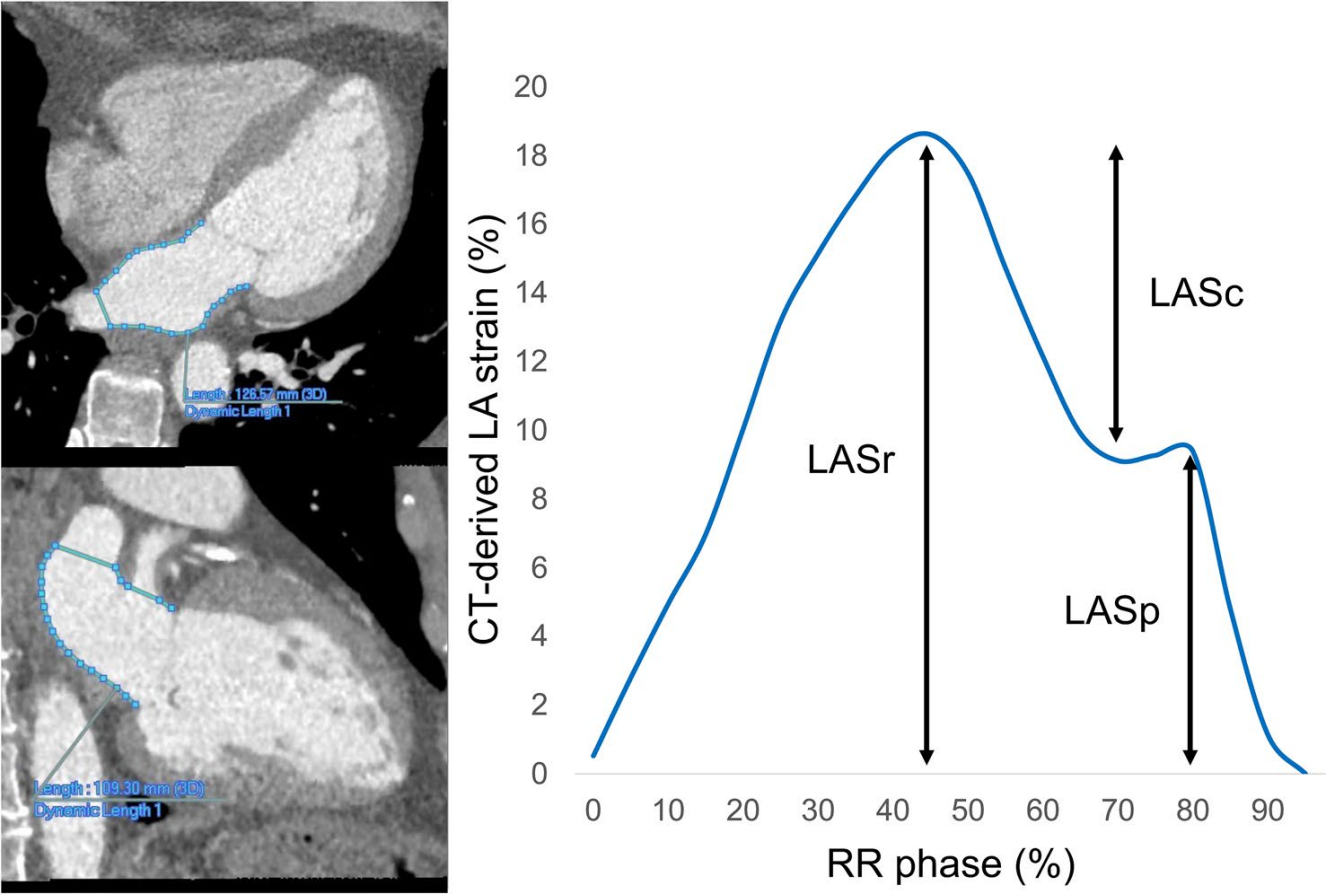
Illustrative examples of CT-derived LA strain analysis. *CT* computed tomography, *LASr* left atrial reservoir strain, *LASc* left atrial conduit strain, *LASp* left atrial booster pump strain

In this study, CT-derived LA strain was analyzed using a dedicated workstation (Ziostation2; Ziosoft Inc., Tokyo, Japan). A radiologist with 8 years of cardiac imaging experience created the cine images of the 4- and 2-chamber views (CVs) using multiplanar reconstruction in all the cases. Modified semi-automatic strain analysis was used for CT-derived LA strain measurement in this study [20] (Fig. 2). A radiologist with 5 years of cardiac imaging experience traced the endocardial borders of the LA in the LV end-diastole in 4- and 2-CVs, excluding the pulmonary veins (PVs) and LA appendage orifices. The contours were subsequently automatically propagated to the other phases of the entire cardiac cycle. In case of inadequate tracking, manual corrections were made when necessary. The zero strain reference was set at LV end-diastole [21]. Three types of CT-derived LA strain were analyzed: reservoir strain (LASr), the peak strain value at the LV end-systole (at the mitral valve opening); booster pump strain (LASp), the strain value at the onset of atrial contraction; and conduit strain (LASc), the difference between LASr and LASp. These were calculated in 4- and 2-CVs, and the averages were used in this study.

For assessing the intra- and inter-observer reliability of CT-derived LA strain, 30 patients were randomly selected (15 patients with HCM and 15 non-HCM patients) from the dataset. The same observer as the first evaluated the CT-derived LA strain in this dataset again at least 1 month after the initial analysis for intra-observer reliability. Another radiologist with 12 years of cardiac imaging experience evaluated CT-derived LA strain in this dataset in the same manner as described above for inter-observer reliability.

#### LA volume and LV mass analysis

CT-derived LA volume and LV mass were analyzed using a dedicated workstation (Synapse Vincent ver. 5; Fujifilm Medical Systems, Tokyo, Japan). We analyzed LA volume in all phases of the cardiac cycle using a three-dimensional image of the LA created by volume rendering, excluding the LA appendage and PVs, and LA maximum volume was measured. The CT-derived LA volume index (LAVI) was calculated by dividing the LA maximum volume by the body surface area (BSA). LV mass was analyzed at a phase in LV end-diastole (RR 0%). LV mass index (LVMI) was calculated by dividing LV mass by BSA.

#### LV longitudinal strain analysis

CT-derived LV longitudinal strain (LVLS) was analyzed in the same 4- and 2-CVs as the LA strain analysis using a dedicated workstation (Ziostation2; Ziosoft Inc., Tokyo, Japan) [20]. The LV mid-wall was traced in LV end-diastole by the same observer as LA strain analysis, and the traces were propagated to the other phases of the entire cardiac cycle. Manual corrections were made when necessary. The zero strain reference was set at LV end-diastole. The negative peak of the strain value at LV end-systole was defined as CT-derived LVLS. The averages of the 4- and 2-CVs were used in this study.

### Statistical analysis

Normality of the data was assessed using the Shapiro–Wilk test. Normally distributed data are presented as mean ± standard deviation, while non-normally distributed data are expressed as median (interquartile range). To compare the data of the two groups, Student’s *t* tests were used for normally distributed data, Mann–Whitney *U* tests were used for non-normally distributed data, and Chi-square tests were used for categorical data. The correlations of CT-derived LA strain with CT-derived LAVI, LVLS, and LVMI were assessed using Pearson’s correlation coefficient or Spearman’s rank correlation coefficient. The intra- and inter-observer reliability for CT-derived LA strain was assessed by the intraclass correlation coefficient (ICC) and Bland–Altman analysis. *p* values < 0.05 were considered statistically significant. Statistical analyses were performed using R statistics version 4.1.3 (R Foundation for Statistical Computing, Vienna, Austria).

## Results

### Study population

Of the 53 patients with HCM, 14 and 5 were excluded owing to non-sinus rhythm at the time of cardiac CT scanning and post-PM or ICD implantation, respectively. Therefore, 34 patients with HCM were eligible for this study. For the non-HCM patients, 31 patients with normal cardiac function were selected [17]. Patients with HCM underwent cardiac CT for coronary artery evaluation for chest pain (*n* = 15), pre-operative evaluation for percutaneous transluminal septal myocardial ablation (PTSMA) (*n* = 9), pre-operative evaluation for catheter ablation for paroxysmal atrial fibrillation (PAF) (*n* = 8), and the others (*n* = 2). Non-HCM patients underwent cardiac CT for coronary artery evaluation for chest pain (*n* = 25) and for abnormal ECG (*n* = 6). The patient characteristics of the HCM and non-HCM patient groups are shown in Table 1. The median age of the patients with HCM was 69.5 years (67.0–75.8 years), and 17 (50%) were male, while the median age of the non-HCM patients was 56.0 years (49.0–63.5 years), and 9 (29%) were male. There were significant differences in the age, hypertension, and family history of cardiac disease between the two groups. Eight (24%) and three (9%) patients with HCM had PAF and moderate mitral regurgitation (MR), respectively. Patients with HCM had significantly lower *e*′ velocities (median 4.2 vs. 6.6 cm/s) and higher *E*/*e*′ (median 15.1 vs. 9.2) than non-HCM patients (*p* < 0.001 in each). Based on echocardiography, 16 (47%) and 10 patients (29%) with HCM were classified into Grade 1 (DD1) and Grade 2 diastolic dysfunction (DD2), respectively; the diastolic dysfunction grade could not be determined in 8 patients (24%) with HCM [18]. All the non-HCM patients had no diastolic dysfunction. The HR, contrast medium dose, and dose length product (DLP) in cardiac CT were 66.0 ± 11.1 bpm, 56.9 ml (52.1–66.2 ml), and 364.8 mGy cm (298.7–466.2 mGy cm), respectively.

**Table 1:** Patient characteristics

	HCM *n* = 34	Non-HCM patients *n* = 31	*p* value^*^
Age (years)	69.5 (67.0–75.8)	56.0 (49.0–63.5)	< 0.001
Sex, male, *n* (%)	17 (50)	9 (29)	0.13
Body mass index (kg/m^2^)	23.5 (22.0–25.5)	22.6 (20.5–27.3)	0.60
Body surface area (m^2^)	1.6 ± 0.2	1.6 ± 0.2	0.46
Risk factors, *n* (%)			
Hypertension	17 (50)	3 (10)	< 0.001
Dyslipidemia	13 (38)	8 (26)	0.42
Diabetes mellitus	6 (18)	4 (13)	0.74
Smoking	12 (35)	8 (26)	0.58
Family history of cardiac disease	18 (53)	8 (26)	0.048
Paroxysmal atrial fibrillation, *n* (%)	8 (24)	0 (0)	0.0052
Mitral regurgitation, *n* (%)			
None—mild	31 (91)	31 (100)	0.24
Moderate	3 (9)	0 (0)	
Echocardiographic parameters			
*E* velocity (cm/s)	70.1 ± 23.7	63.8 ± 15.5	0.22
*A* velocity (cm/s)	78.6 ± 26.6	67.9 ± 17.3	0.067
*e*′ velocity (cm/s)	4.2 (3.4–5.2)	6.6 (5.8–8.2)	< 0.001
*E*/*A* ratio	0.9 (0.7–1.0)	0.9 (0.7–1.1)	0.47
*E*/*e*′ ratio	15.1 (10.4–20.0)	9.2 (8.3–10.2)	< 0.001
LVEDV (ml)	64.4 ± 16.5	65.4 ± 17.7	0.81
LVESV (ml)	24.3 (14.6–27.9)	21.8 (18.1–25.7)	0.67
LVEF (%)	63.7 ± 8.4	65.7 ± 5.7	0.28
Diastolic dysfunction grade			
DD1	16 (47%)		
DD2	10 (29%)		
Indeterminate	8 (24%)		

Data are presented as the mean ± standard deviation, median (25th–75th percentile), or the number (%) of participants

*HCM* hypertrophic cardiomyopathy, *LVEDV* left ventricular end-diastolic volume, *LVESV* left ventricular end-systolic volume, *LVEF* left ventricular ejection fraction, *LAVI* left atrial volume index, *DD1* Grade 1 diastolic dysfunction, *DD2* Grade 2 diastolic dysfunction

*p value compared with patients with HCM and non-HCM patients

### Relations between CT-derived LA strain and conventional functional indices in patients with HCM

In patients with HCM, the LASr, LASc, and LASp were 20.8 ± 7.6%, 7.9 ± 3.4%, and 12.8 ± 5.7%, respectively. CT-derived LAVI, LVLS, and LVMI were 103.0 ± 32.7 ml/m^2^, − 13.4 ± 4.3%, and 71.1 g/m^2^ (64.4–85.6 g/m^2^), respectively.

**Fig. 3:**
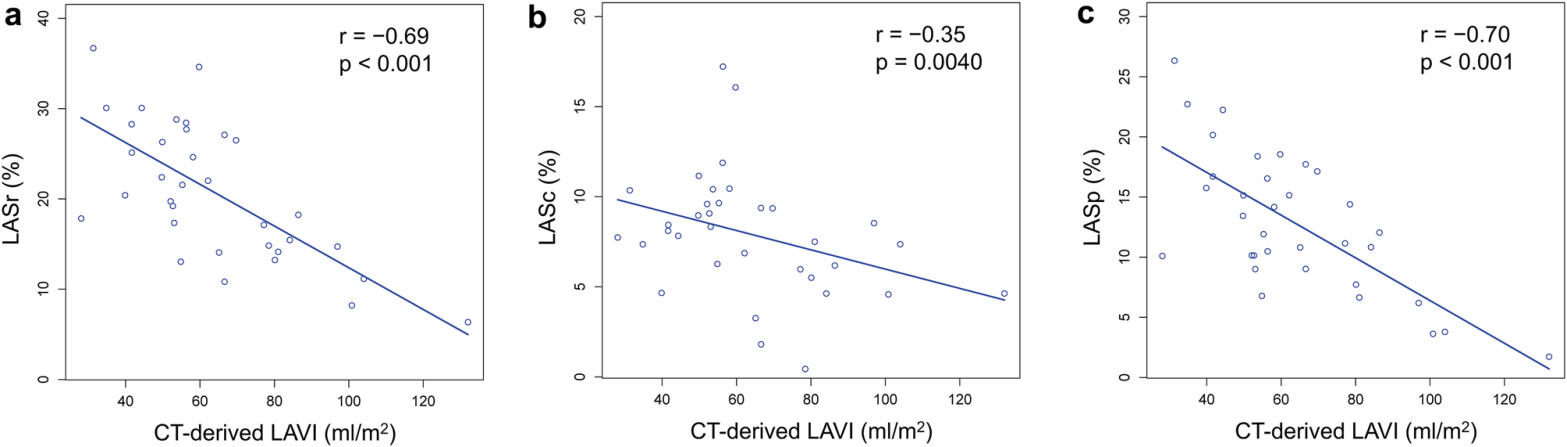
Correlations between CT-derived LA strain and LAVI. There are significant correlations between CT-derived LA strain [LASr (**a**), LASc (**b**), LASp (**c**)] and LAVI. *CT* computed tomography, *LAVI* left atrial volume index, *LASr* left atrial reservoir strain, *LASc* left atrial conduit strain, *LASp* left atrial booster pump strain

**Fig. 4:**
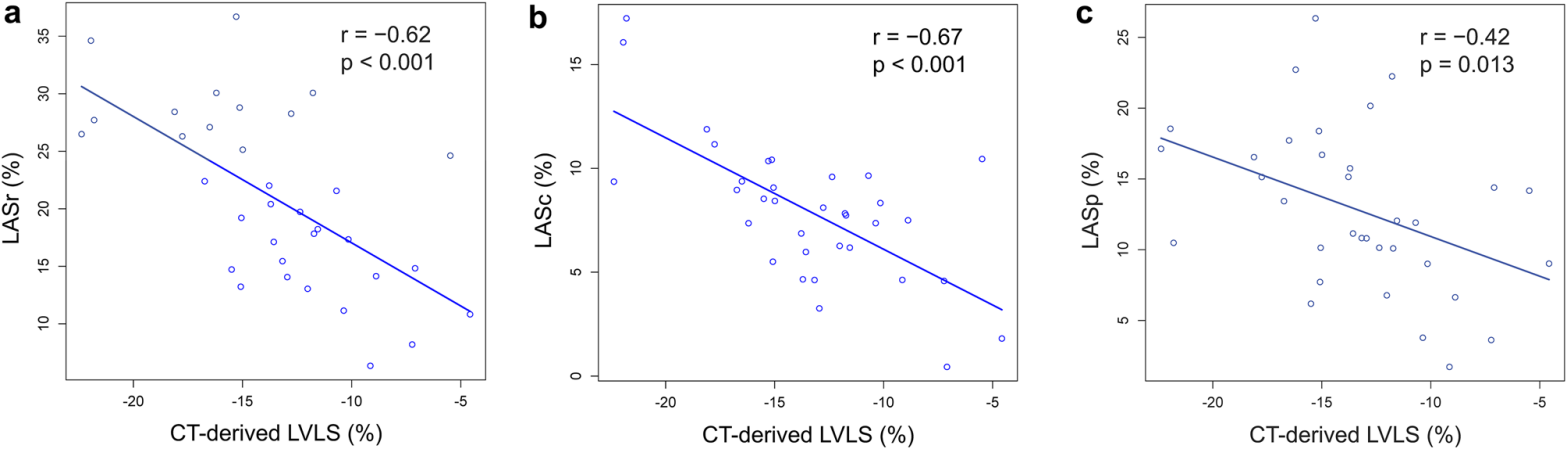
Correlations between CT-derived LA strain and LVLS. There are significant correlations between CT-derived LA strain [LASr (**a**), LASc (**b**), LASp (**c**)] and LVLS. *CT* computed tomography, *LVLS* left ventricular longitudinal strain, *LASr* left atrial reservoir strain, *LASc* left atrial conduit strain, *LASp* left atrial booster pump strain

A significant negative correlation was observed between the CT-derived LA strain and LAVI, with moderate correlations for LASr (*r* = − 0.69, *p* < 0.001) and LASp (*r* = − 0.70, *p* < 0.001), and a weak correlation for LASc (*r* = − 0.35, *p* = 0.0040) (Fig. 3). A significant negative correlation was also observed between CT-derived LA strain and LVLS, with moderate correlations for LASr (*r* = − 0.62, *p* < 0.001) and LASc (*r* = − 0.67, *p* < 0.001), and a weak correlation for LASp (*r* = − 0.42, *p* = 0.013) (Fig. 4). CT-derived LVMI showed significant correlations with LASr (*ρ* = − 0.45, *p* = 0.0077) and LASc (*ρ* = − 0.48, *p* = 0.0043), while it was not significantly correlated with LASp.

In patients with HCM with DD1, no significant correlation was observed between CT-derived LA strain and LAVI. In patients with HCM with DD2, LASr and LASp significantly correlated with CT-derived LAVI (*r* = − 0.81, *p* = 0.0048; and *r* = − 0.86, *p* = 0.0016; respectively), whereas LASc was not significantly correlated with CT-derived LAVI.

### Comparison between patients with HCM and non-HCM patients in CT-derived LA strain, LAVI, LVLS, and LVMI

**Fig. 5:**
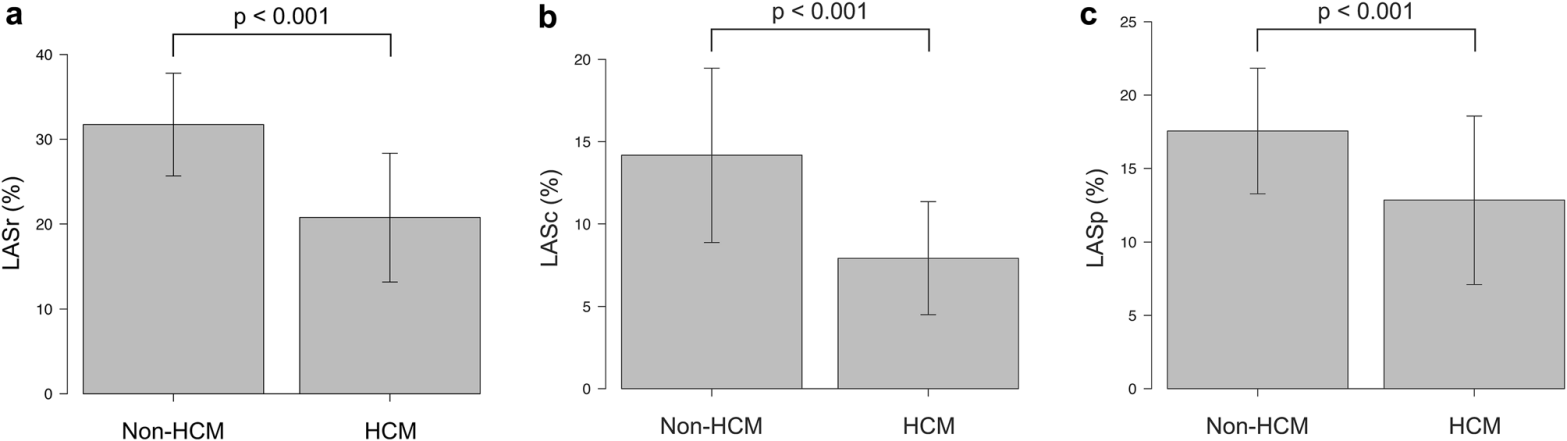
Comparison of CT-derived LA strain between patients with HCM and non-HCM patients. CT-derived LA strain [LASr (**a**), LASc (**b**), LASp (**c**)] was significantly lower in patients with HCM than those in non-HCM patients. *HCM* hypertrophic cardiomyopathy, *CT* computed tomography, *LASr* left atrial reservoir strain, *LASc* left atrial conduit strain, *LASp* left atrial pump strain

In the non-HCM patients, LASr, LASc, and LASp were 31.7 ± 6.1%, 14.2 ± 5.3%, and 17.6 ± 4.3%, respectively. LASr, LASc, and LASp were significantly lower in patients with HCM than those in the non-HCM patients (*p* < 0.001, in each) (Fig. 5). CT-derived LAVI, LVLS, and LVMI were 63.7 ± 22.7 ml/m^2^, − 21.4 ± 2.9%, and 39.9 g/m^2^ (35.5–48.7 g/m^2^), respectively. CT-derived LAVI and LVMI were significantly larger in patients with HCM than in non-HCM patients (*p* < 0.001), and CT-derived LVLS was significantly decreased in patients with HCM than in non-HCM patients (*p* < 0.001). These results are shown in Table 2.

**Table 2:** CT-derived LA and LV functional parameters

	HCM *n* = 34	Non-HCM patients *n* = 31	*p* value*
CT-derived LA strain			
LASr (%)	20.8 ± 7.6	31.7 ± 6.1	< 0.001
LASc (%)	7.9 ± 3.4	14.2 ± 5.3	< 0.001
LASp (%)	12.8 ± 5.7	17.6 ± 4.3	< 0.001
CT-derived LAVI (ml/m^2^)	103.0 ± 32.7	63.7 ± 22.7	< 0.001
CT-derived LVLS (%)	− 13.4 ± 4.3	− 21.4 ± 2.9	< 0.001
CT-derived LV mass (g)	116.1 (106.1–148.6)	66.2 (57.0–78.5)	< 0.001
CT-derived LVMI (g/m^2^)	71.1 (64.4–85.6)	39.9 (35.5–48.7)	< 0.001

Data are presented as the mean ± standard deviation, median (25th–75th percentile)

*p value compared with patients with HCM and non-HCM patients

*CT* computed tomography, *HCM* hypertrophic cardiomyopathy, *LASr* left atrial reservoir strain, *LASc* left atrial conduit strain, *LASp* left atrial booster pump strain, *LAVI* left atrial volume index, *LVLS* left ventricular global longitudinal strain, *LVMI* left ventricular mass index

### Reproducibility of CT-derived LA strain assessment

**Fig. 6:**
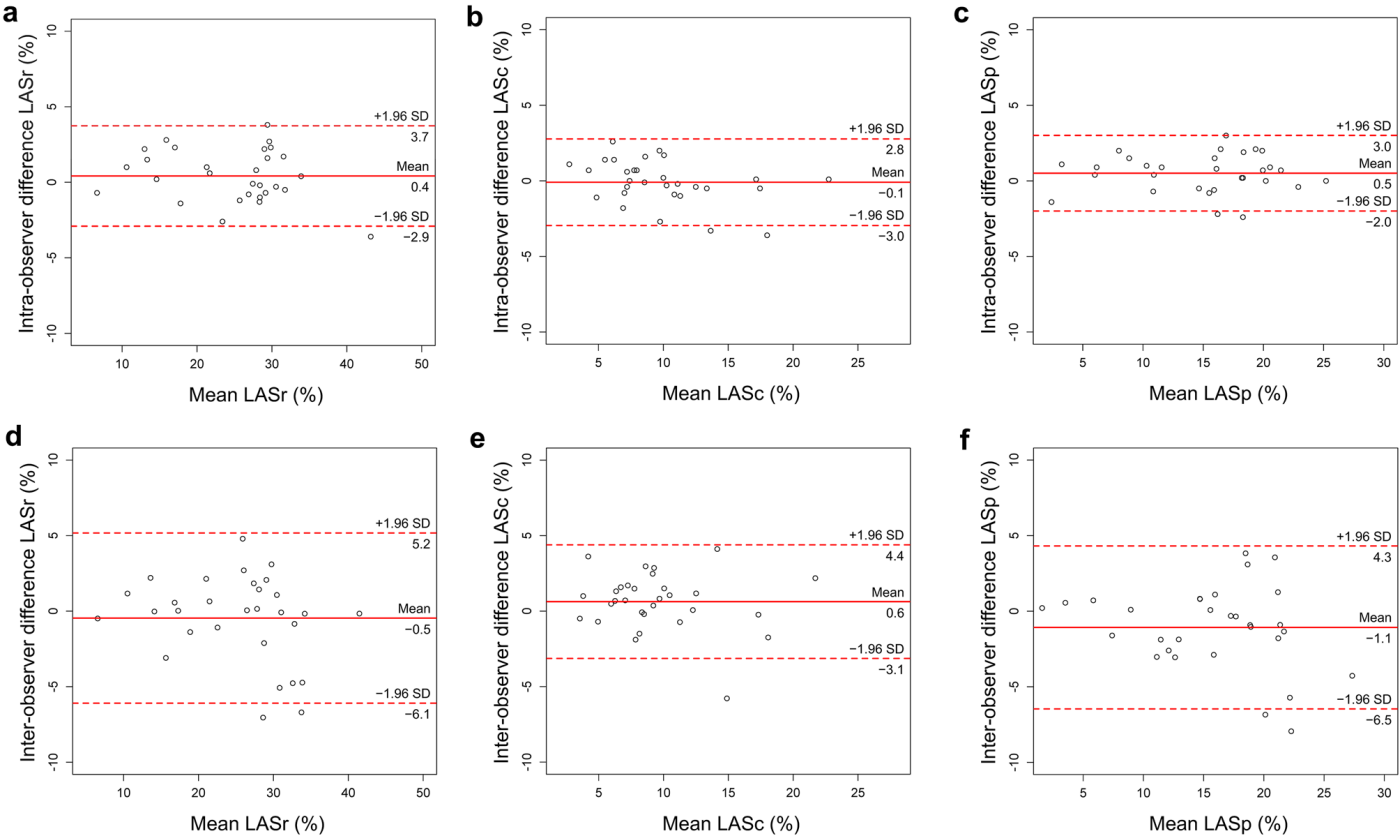
Bland–Altman plots for inter- and intra-observer analysis. Bland–Altman plots of LASr, LASc, and LASp for intra- (**a**–**c**) and inter-observer analysis (**d**–**f**). *LASr* left atrial reservoir strain, *LASc* left atrial conduit strain, *LASp* left atrial pump strain

The CT-derived LA strain showed high reproducibility at both intra- and inter-observer levels. The ICCs for LASr, LASc, and LASp were 0.98 (95% Confidence interval [CI] 0.95–0.99), 0.97 (95% CI 0.95–0.99), and 0.95 (95% CI 0.90–0.98) for intra-observer, and 0.94 (95% CI 0.88–0.97), 0.90 (95% CI 0.80–0.95), and 0.89 (95% CI 0.77–0.95) for inter-observer levels, respectively. The Bland–Altman analysis for LASr, LASc, and LASp showed mean differences of 0.4% (95% CI − 2.9 to 3.7%), − 0.1% (95% CI − 3.0 to 2.8%), and 0.5% (95% CI − 2.0 to 3.0%) for intra-observer, and − 0.5% (95% CI − 6.1 to 5.2%), 0.6% (95% CI − 3.1 to 4.4%), and − 1.1% (95% CI − 6.5 to 4.3%) for inter-observer levels, respectively (Fig. 6).

## Discussion

In this study, we have demonstrated that: (1) CT-derived LA strain had significant correlations with CT-derived LAVI and LVLS in patients with HCM, (2) CT-derived LA strain was significantly lower in patients with HCM than that in non-HCM patients, and (3) CT-derived LA strain assessment is highly reproducible.

A significant correlation was observed between CT-derived LA strain (LASr, LASc, and LASp) and LAVI. In patients with HCM, the LV filling pressure and LV end-diastolic pressure are elevated, resulting in elevated LA pressure [2]. Then, the elevated LA pressure causes remodeling of the LA myocardium leading to LA enlargement and decreased compliance [2]. Additionally, the elevated LA pressure causes a decreased PV return. LA reservoir function is affected by the decreased LA compliance and PV return, resulting in decreased LASr [22]. LV filling pressure is an afterload for LA conduit function; thus, the elevated LV filling pressure is associated with a decrease in LASc [23]. The LA booster pump function is affected by the LV end-diastolic pressure and PV return [24]. Elevated LV end-diastolic pressure indicates an increase in the afterload, while a reduced PV return indicates a decrease in the preload, resulting in a decrease in LASp [4]. Thus, we consider that the CT-derived LA strain and LAVI showed a significant correlation since the pathophysiology is common to LA strain and LAVI. In patients with mild LV diastolic dysfunction, the enlargement of LAVI is absent or mild, whereas LASr and LASc are already decreased, indicating a discrepancy between LAVI and LA strain changes [7, 23]. Indeed, there were no significant correlations between CT-derived LA strain and LAVI in patients with HCM with DD1. On the other hand, in patients with moderate or severe LV diastolic dysfunction, LAVI enlarges and CT-derived LA strain decreases with the progression of LV diastolic dysfunction. However, previous reports have demonstrated that conduit function is decreased to a lesser extent between DD1 and DD2 with decreased booster pump function [7, 23, 25]. In this study, LASr and LASp strongly correlated with CT-derived LAVI, while LASc did not correlate with CT-derived LAVI in patients with HCM with DD2. Therefore, the correlation between CT-derived LA strain and LAVI may mainly reflect the changes in DD2, and LASc showed relatively weak correlation with CT-derived LAVI.

Significant correlations were also observed between CT-derived LA strain and LVLS. Although HCM is associated with LV diastolic dysfunction, it also causes LV systolic dysfunction owing to LV hypertrophy and myocardial fibrosis [26, 27]. Thus, LVLS, an index of LV contractility, is also decreased in HCM. The LA reservoir function is closely related to the descending movement of the LV base during LV systole [28, 29]. LA conduit function refers to the passive emptying of the LA by LV dilatation. Impaired LV systolic function in HCM causes a decrease in elastic recoil, which leads to LV diastolic dysfunction in the LV early diastole [30]. Hence, we suspect that LASr and LASc showed significant correlations with CT-derived LVLS in this study. Since the LA booster pump function is affected by the active contractility of the LA and LV end-diastolic pressure, LASp is less directly affected by the LV systolic function, resulting in a relatively weak correlation between LASp and CT-derived LVLS in this study. However, fibrosis and hypertrophy of LV myocardium are associated with both LV systolic and diastolic dysfunction, leading to the decrease of both LASp and CT-derived LVLS with the indirect relationship.

CT-derived LVMI significantly correlated with LASr and LASc. Previous studies have shown that LVMI correlates with LV global longitudinal strain, reflecting myocardial disarray and fibrosis [31]. Thus, we suspect that similar results were observed for LVMI and LVLS.

All the CT-derived LA strain values were significantly lower in patients with HCM than in the non-HCM patients. Considering the CT-derived LA strain in the non-HCM patients, LASr and LASc in the present study tended to be lower than the normal reference values in those measured with STE, while LASp showed a similar value [10, 32]. Similar trends have been observed in previous studies, possibly owing to the lower temporal resolution of cardiac CT than that of echocardiography [11, 12]. In patients with low heart rates, LV mid-diastole is the longest quiescent phase in the cardiac cycle, followed by LV end-systole. LASp is the peak strain at LV mid-diastole, and LASr is the peak strain at LV end-systole. In this study, the patient’s HR was low enough (mean 66.0 bpm) to have a sufficient quiescent phase in the LV mid-diastole to capture the true peak of LASp, while the true peak of LASr was less likely to be captured owing to insufficient temporal resolution of cardiac CT [33]. In addition, LV stiffening with age causes a decrease in LASr and LASc and an increase in LASp [10, 32]. The non-HCM patients in this study were relatively older than those in previous reports [10, 32]. When referring to the LA strain values in the group of similar ages, the CT-derived LA strain values in this study were closer to those measured with STE in the previous studies [10, 32]. Additionally, cardiac CT is performed under inspiratory breath-hold with contrast injection, while echocardiography is performed under free-breathing without contrast injection, which may have affected the LA strain values [34, 35]. Thus, these influences should be considered for the interpretation of CT-derived LA strain in this study. Nevertheless, we selected patients with normal cardiac function (as far as we could confirm), and CT used in this study had the highest temporal resolution currently available (66 ms) [17]. Therefore, we believe the CT-derived LA strain values in non-HCM patients to be reasonable in this study. Despite these conditions, CT-derived LA strain in patients with HCM was even lower than those in non-HCM patients, suggesting CT-derived LA strain reflected LA dysfunction in patients with HCM.

In the present study, the CT-derived LA strain showed high reproducibility as shown in the previous studies [11, 12]. Furthermore, the CT-derived LA strain could be analyzed in all the cases in this study. Although STE is the most widely used method for LA strain evaluation, its evaluation was challenging in some cases owing to the low image quality [10–12]. Cardiac MRI is another modality used for LA strain assessment, but some patients cannot be examined due to contraindications [8]. Cardiac CT is often performed for the evaluation of coronary artery disease and pre-operative assessment for PTSMA, and is available for cardiac functional evaluation comparable to MRI [13, 36]. Especially regarding LA volume, which is one of the conventional quantitative indices of LA function, cardiac CT allows a three-dimensional assessment, which is more accurate than the modified Simpson method used in echocardiography and MRI [14]. Therefore, when cardiac CT is performed with retrospective ECG-gated mode for any clinical indications, CT-derived LA strain and LA volume analysis could be an alternative for the quantitative assessment of LA function in patients with HCM.

This study has several limitations. First, this was a single-center retrospective study with a relatively small sample size. Therefore, we could not evaluate the CT-derived LA strain in HCM types, such as HOCM or non-obstructive HCM, although LA dysfunction is more severe in HOCM [37]. Second, some patients in the HCM group had PAF or MR. The development of AF and reduced LA strain are interrelated; patients with reduced LA strain are more likely to develop AF, and patients with AF are more likely to have lower LA strain [38, 39]. MR is also related to LA dysfunction owing to the LA volume overload [2]. Additionally, medications possibly affected the LA function in patients with HCM. These could have influenced the decrease in the LA strain in the HCM group. Third, since we selected non-HCM patients based on strict criteria, we could not match the patients’ backgrounds (e.g., age and hypertension) between patients with HCM and non-HCM patients due to the small study population, which might have affected the results. Fourth, although STE is the most common method for LA strain evaluation, we did not compare the strain values between CT- and STE-derived LA strain in this study. The STE-derived LA strain values are affected by the vendor of the echocardiography and analyzing software [40]. In this study, both patients with HCM and non-HCM patients underwent echocardiography from several vendors. Previous studies have shown moderate to strong correlations between CT- and STE-derived LA strain in other populations [11, 12]. We suppose that similar results would come out in patients with HCM. However, further studies are needed to confirm this. Fifth, although we used the best temporal resolution CT scanner currently available (66 ms, about 15 fps) in this study, it is lower than that of STE (50–70 fps), which might have affected the results [4]. Previous studies have shown that CT-derived LA strain is relatively lower than STE [11, 12]. Therefore, the CT-derived LA strain values should be interpreted with caution. Sixth, although CT-derived LA strain analysis showed high reproducibility in both intra- and inter-observer levels based on the ICCs, the Bland–Altman analysis showed that the inter-observer difference range was not small. In this study, we analyzed CT-derived LA strain semi-automatically using a dedicated workstation; however, the development of analyzing software including full automation is needed to further improve the reproducibility. Lastly, radiation exposure was relatively high since the cardiac CT was scanned with retrospective ECG-gated mode for several reasons in this study. For the pre-operative evaluation for PTSMA, retrospective ECG-gated mode was used to identify the target myocardium in LV systole and feeding septal branch in LV diastole [36]. Patients with HCM, except for those with PAF, were the participants in another prospective study, and written informed consent was obtained for cardiac CT with retrospective ECG-gated mode. For patients with PAF and non-HCM patients, retrospective ECG-gated mode was used when the attending physician considered it necessary to evaluate cardiac structure and function. Since the use of retrospective ECG-gated mode was associated with higher radiation exposure, it was not routinely used for coronary CT in our institution. However, if retrospective ECG-gated mode was needed, we used various techniques, such as low tube voltage scan, dose modulation, and iterative reconstruction, to reduce radiation exposure as much as possible. As a result, the radiation exposure associated with cardiac CT was less than one-third of the Japanese diagnostic reference level (DRL) (1300 mGy cm) and less than international DRL (400 mGy cm) [41, 42]. Nevertheless, further reduction of radiation exposure is desirable.

## Conclusion

CT-derived LA strain is feasible for quantitative assessment of LA function with high reproducibility and could be an alternative method for assessing LA function in patients with HCM.

## References

[1] Semsarian C, Ingles J, Maron MS, Maron BJ (2015). New perspectives on the prevalence of hypertrophic cardiomyopathy. J Am Coll Cardiol.

[2] Abhayaratna WP, Seward JB, Appleton CP, Douglas PS, Oh JK, Tajik AJ (2006). Left atrial size: physiologic determinants and clinical applications. J Am Coll Cardiol.

[3] Ohsato K, Shimizu M, Sugihara N, Konishi K, Takeda R (1992). Histopathological factors related to diastolic function in myocardial hypertrophy. Jpn Circ J.

[4] Hoit BD (2014). Left atrial size and function: role in prognosis. J Am Coll Cardiol.

[5] Maron BJ, Ommen SR, Semsarian C, Spirito P, Olivotto I, Maron MS (2014). Hypertrophic cardiomyopathy: present and future, with translation into contemporary cardiovascular medicine. J Am Coll Cardiol.

[6] Debonnaire P, Joyce E, Hiemstra Y, Mertens BJ, Atsma DE, Schalij MJ (2017). Left atrial size and function in hypertrophic cardiomyopathy patients and risk of new-onset atrial fibrillation. Circ Arrhythm Electrophysiol.

[7] Brecht A, Oertelt-Prigione S, Seeland U, Rucke M, Hattasch R, Wagelohner T (2016). Left atrial function in preclinical diastolic dysfunction: two-dimensional speckle-tracking echocardiography-derived results from the BEFRI trial. J Am Soc Echocardiogr.

[8] Yang Y, Yin G, Jiang Y, Song L, Zhao S, Lu M (2020). Quantification of left atrial function in patients with non-obstructive hypertrophic cardiomyopathy by cardiovascular magnetic resonance feature tracking imaging: a feasibility and reproducibility study. J Cardiovasc Magn Reson.

[9] Jain V, Ghosh R, Gupta M, Saijo Y, Bansal A, Farwati M (2021). Contemporary narrative review on left atrial strain mechanics in echocardiography: cardiomyopathy, valvular heart disease and beyond. Cardiovasc Diagn Ther.

[10] Pathan F, D'Elia N, Nolan MT, Marwick TH, Negishi K (2017). Normal ranges of left atrial strain by speckle-tracking echocardiography: a systematic review and meta-analysis. J Am Soc Echocardiogr.

[11] Szilveszter B, Nagy AI, Vattay B, Apor A, Kolossváry M, Bartykowszki A (2020). Left ventricular and atrial strain imaging with cardiac computed tomography: validation against echocardiography. J Cardiovasc Comput Tomogr.

[12] Bernhard B, Grogg H, Zurkirchen J, Demirel C, Hagemeyer D, Okuno T (2022). Reproducibility of 4D cardiac computed tomography feature tracking myocardial strain and comparison against speckle-tracking echocardiography in patients with severe aortic stenosis. J Cardiovasc Comput Tomogr.

[13] Rizvi A, Deano RC, Bachman DP, Xiong G, Min JK, Truong QA (2015). Analysis of ventricular function by CT. J Cardiovasc Comput Tomogr.

[14] Badano LP, Miglioranza MH, Mihaila S, Peluso D, Xhaxho J, Marra MP (2016). Left atrial volumes and function by three-dimensional echocardiography: reference values, accuracy, reproducibility, and comparison with two-dimensional echocardiographic measurements. Circ Cardiovasc Imaging.

[15] Hirasawa K, Kuneman JH, Singh GK, Gegenava T, Hautemann D, Reiber JHC (2021). Comparison of left atrial strain measured by feature tracking computed tomography and speckle tracking echocardiography in patients with aortic stenosis. Eur Heart J Cardiovasc Imaging.

[16] Kitaoka H, Tsutsui H, Kubo T, Ide T, Chikamori T, Fukuda K (2021). JCS/JHFS 2018 guideline on the diagnosis and treatment of cardiomyopathies. Circ J.

[17] Yoshida K, Tanabe Y, Kido T, Kurata A, Uraoka D, Kinoshita M (2020). Characteristics of the left ventricular three-dimensional maximum principal strain using cardiac computed tomography: reference values from subjects with normal cardiac function. Eur Radiol.

[18] Nagueh SF, Smiseth OA, Appleton CP, Byrd BF, Dokainish H, Edvardsen T (2016). Recommendations for the evaluation of left ventricular diastolic function by echocardiography: an update from the American Society of Echocardiography and the European Association of Cardiovascular Imaging. J Am Soc Echocardiogr.

[19] Kawaguchi N, Kurata A, Kido T, Nishiyama Y, Kido T, Miyagawa M (2013). Optimization of coronary attenuation in coronary computed tomography angiography using diluted contrast material. Circ J.

[20] Ammon F, Bittner D, Hell M, Mansour H, Achenbach S, Arnold M (2019). CT-derived left ventricular global strain: a head-to-head comparison with speckle tracking echocardiography. Int J Cardiovasc Imaging.

[21] Badano LP, Kolias TJ, Muraru D, Abraham TP, Aurigemma G, Edvardsen T (2018). Standardization of left atrial, right ventricular, and right atrial deformation imaging using two-dimensional speckle tracking echocardiography: a consensus document of the EACVI/ASE/Industry Task Force to standardize deformation imaging. Eur Heart J Cardiovasc Imaging.

[22] Appleton CP (1997). Hemodynamic determinants of doppler pulmonary venous flow velocity components: new insights from studies in lightly sedated normal dogs. J Am Coll Cardiol.

[23] Otani K, Takeuchi M, Kaku K, Haruki N, Yoshitani H, Tamura M (2010). Impact of diastolic dysfunction grade on left atrial mechanics assessed by two-dimensional speckle tracking echocardiography. J Am Soc Echocardiogr.

[24] Nagueh SF, Sun H, Kopelen HA, Middleton KJ, Khoury DS (2001). Hemodynamic determinants of the mitral annulus diastolic velocities by tissue Doppler. J Am Coll Cardiol.

[25] Anwar AM, Geleijnse ML, Soliman OI, Nemes A, ten Cate FJ (2007). Left atrial Frank-Starling law assessed by real-time, three-dimensional echocardiographic left atrial volume changes. Heart.

[26] Almaas VM, Haugaa KH, Strom EH, Scott H, Smith HJ, Dahl CP (2014). Noninvasive assessment of myocardial fibrosis in patients with obstructive hypertrophic cardiomyopathy. Heart.

[27] Haland TF, Hasselberg NE, Almaas VM, Dejgaard LA, Saberniak J, Leren IS (2017). The systolic paradox in hypertrophic cardiomyopathy. Open Heart.

[28] Barbier P, Solomon SB, Schiller NB, Glantz SA (1999). Left atrial relaxation and left ventricular systolic function determine left atrial reservoir function. Circulation.

[29] Liu H, Pozios I, Haileselassie B, Nowbar A, Sorensen LL, Phillip S (2017). Role of global longitudinal strain in predicting outcomes in hypertrophic cardiomyopathy. Am J Cardiol.

[30] Gilbert JC, Glantz SA (1989). Determinants of left ventricular filling and of the diastolic pressure-volume relation. Circ Res.

[31] Urbano-Moral JA, Rowin EJ, Maron MS, Crean A, Pandian NG (2014). Investigation of global and regional myocardial mechanics with 3-dimensional speckle tracking echocardiography and relations to hypertrophy and fibrosis in hypertrophic cardiomyopathy. Circ Cardiovasc Imaging.

[32] Nielsen AB, Skaarup KG, Hauser R, Johansen ND, Lassen MCH, Jensen GB (2021). Normal values and reference ranges for left atrial strain by speckle-tracking echocardiography: the Copenhagen City Heart Study. Eur Heart J Cardiovasc Imaging.

[33] Seifarth H, Wienbeck S, Pusken M, Juergens KU, Maintz D, Vahlhaus C (2007). Optimal systolic and diastolic reconstruction windows for coronary CT angiography using dual-source CT. AJR Am J Roentgenol.

[34] Reiter C, Reiter U, Kräuter C, Nizhnikava V, Greiser A, Scherr D (2021). Differences in left ventricular and left atrial function assessed during breath-holding and breathing. Eur J Radiol.

[35] Gottfridsson P, A’Roch R, Lindqvist P, Law L, Myberg T, Hultin M (2022). Left atrial contraction strain and controlled preload alterations, a study in healthy individuals. Cardiovasc Ultrasound.

[36] Cooper RM, Binukrishnan SR, Shahzad A, Hasleton J, Sigwart U, Stables RH (2017). Computed tomography angiography planning identifies the target vessel for optimum infarct location and improves clinical outcome in alcohol septal ablation for hypertrophic obstructive cardiomyopathy. EuroIntervention.

[37] Williams LK, Chan RH, Carasso S, Durand M, Misurka J, Crean AM (2015). Effect of left ventricular outflow tract obstruction on left atrial mechanics in hypertrophic cardiomyopathy. Biomed Res Int.

[38] Kawakami H, Ramkumar S, Nolan M, Wright L, Yang H, Negishi K (2019). Left Atrial mechanical dispersion assessed by strain echocardiography as an independent predictor of new-onset atrial fibrillation: a case–control study. J Am Soc Echocardiogr.

[39] Yoon YE, Kim HJ, Kim SA, Kim SH, Park JH, Park KH (2012). Left atrial mechanical function and stiffness in patients with paroxysmal atrial fibrillation. J Cardiovasc Ultrasound.

[40] Pathan F, Zainal Abidin HA, Vo QH, Zhou H, D'Angelo T, Elen E (2021). Left atrial strain: a multi-modality, multi-vendor comparison study. Eur Heart J Cardiovasc Imaging.

[41] Tanabe Y, Kido T, Kimura F, Kobayashi Y, Matsunaga N, Yoshioka K (2020). Japanese survey of radiation dose associated with coronary computed tomography angiography—2013 data from a multicenter registry in daily practice. Circ J.

[42] Stocker TJ, Deseive S, Leipsic J, Hadamitzky M, Chen MY, Rubinshtein R (2018). Reduction in radiation exposure in cardiovascular computed tomography imaging: results from the PROspective multicenter registry on radiaTion dose Estimates of cardiac CT angIOgraphy iN daily practice in 2017 (PROTECTION VI). Eur Heart J.

